# Oral and Fecal *Campylobacter concisus* Strains Perturb Barrier Function by Apoptosis Induction in HT-29/B6 Intestinal Epithelial Cells

**DOI:** 10.1371/journal.pone.0023858

**Published:** 2011-08-24

**Authors:** Hans Linde Nielsen, Henrik Nielsen, Tove Ejlertsen, Jørgen Engberg, Dorothee Günzel, Martin Zeitz, Nina A. Hering, Michael Fromm, Jörg-Dieter Schulzke, Roland Bücker

**Affiliations:** 1 Department of Infectious Diseases, Aalborg Hospital, Aarhus University Hospital, Aalborg, Denmark; 2 Department of Clinical Microbiology, Aalborg Hospital, Aarhus University Hospital, Aalborg, Denmark; 3 Department of Clinical Microbiology, Slagelse Hospital, Slagelse, Denmark; 4 Institute of Clinical Physiology, Charité Universitätsmedizin Berlin, Berlin, Germany; 5 Department of Gastroenterology, Infectious Diseases and Rheumatology, Division of General Medicine and Nutrition, Charité Universitätsmedizin Berlin, Berlin, Germany; University of South Florida College of Medicine, United States of America

## Abstract

*Campylobacter concisus* infections of the gastrointestinal tract can be accompanied by diarrhea and inflammation, whereas colonization of the human oral cavity might have a commensal nature. We focus on the pathophysiology of *C. concisus* and the effects of different clinical oral and fecal *C. concisus* strains on human HT-29/B6 colon cells. Six oral and eight fecal strains of *C. concisus* were isolated. Mucus-producing HT-29/B6 epithelial monolayers were infected with the *C. concisus* strains. Transepithelial electrical resistance (R^t^) and tracer fluxes of different molecule size were measured in Ussing chambers. Tight junction (TJ) protein expression was determined by Western blotting, and subcellular TJ distribution was analyzed by confocal laser-scanning microscopy. Apoptosis induction was examined by TUNEL-staining and Western blot of caspase-3 activation. All strains invaded confluent HT-29/B6 cells and impaired epithelial barrier function, characterized by a time- and dose-dependent decrease in R^t^ either after infection from the apical side but even more from the basolateral compartment. TJ protein expression changes were sparse, only in apoptotic areas of infected monolayers TJ proteins were redistributed. Solely the barrier-forming TJ protein claudin-5 showed a reduced expression level to 66±8% (*P*<0.05), by expression regulation from the gene. Concomitantly, Lactate dehydrogenase release was elevated to 3.1±0.3% versus 0.7±0.1% in control (*P*<0.001), suggesting cytotoxic effects. Furthermore, oral and fecal *C. concisus* strains elevated apoptotic events to 5-fold. *C. concisus*-infected monolayers revealed an increased permeability for 332 Da fluorescein (1.74±0.13 vs. 0.56±0.17 10^−6^ cm/s in control, *P*<0.05) but showed no difference in permeability for 4 kDa FITC-dextran (FD-4). The same was true in camptothecin-exposed monolayers, where camptothecin was used for apoptosis induction.

In conclusion, epithelial barrier dysfunction by oral and fecal *C. concisus* strains could mainly be assigned to apoptotic leaks together with moderate TJ changes, demonstrating a leak-flux mechanism that parallels the clinical manifestation of diarrhea.

## Introduction

*Campylobacter concisus*, first isolated from the human oral cavity, has been proposed as an emerging human enteric pathogen [1]. In contrast to other *Campylobacter* species no primary animal reservoir has been found for *C. concisus*, although it has been detected in diarrheic fecal samples from domestic dogs [2]. Recently, Zhang and coworkers found a very high prevalence of *C. concisus* in saliva from healthy individuals and patients with Crohn's disease suggesting it is an oral commensal rather than a clinical significant oral pathogen [3]. A potential role of emerging *C. concisus* in human gastrointestinal disease has been proposed, and recent studies describe a high incidence of *C. concisus* in pediatric diarrheic stool samples as well as in immunocompromised patients with diarrhea in a tertiary hospital setting [1], [4], [5]. However, the overall prevalence of *C. concisus* in feces is underreported because an easy-to-handle standardized selective culture medium is not available and the time-consuming filter method is not included in standard diagnostics. In a mouse model *C. concisus* induced weight loss and microabscesses in the liver [6], whereas there is only sparse data on the pathophysiology when the microorganism is introduced to the human intestine. *C. concisus* possesses virulence factors including secreted and cell-bound hemolytic activities as well as several putative virulence factors e.g. cytolethal distending toxin [7], [8]. Recently, *C. concisus* genes coding for a presumed Zonula occludens toxin (ZOT) as well as a surface-layer protein belonging to the RTX (repeats in toxin) toxins has been identified [8]. Even though these toxins are recognized as important virulence factors their pathogenic role in *C. concisus* infection is unknown. In a recent work by Kalischuk and Inglis the ZOT gene was detected more often in *C. concisus* isolates from healthy individuals than in isolates from diarrheic humans [9]. In consistence with earlier reports [10], the authors identified two main genetically distinct clusters which differed with respect to their pathogenic properties. ALFP cluster 2 isolates were predominantly found in diarrheic patients and showed higher ability in epithelial invasion and translocation. In contrary, *C. concisus* ALFP cluster 1 were mainly found in fecal isolates from healthy individuals and are present in the oral reference strain ATCC 33237. These strains harbor hemolytic activity and can induce apoptotic DNA fragmentation. Man and coworkers reported recently that *C. concisus* is able to attach to and invade intestinal epithelial cells, and moreover *C. concisus* was observed to invade adjacent to the tight junction (TJ) [11]. In the current study we focus on the pathophysiology of *C. concisus* and describe the effects on epithelial barrier function of different oral and fecal clinical *C. concisus* strains, using the human colon cell line HT-29/B6.

## Results

**Figure 1:**
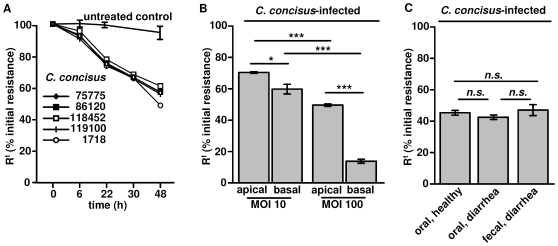
Effect of *Campylobacter concisus* on epithelial barrier function. *(* ***A*** *)* Different *Campylobacter concisus* strains on HT-29/B6 cells. Time-dependent decrease in transepithelial electrical resistance (R^t^). *(* ***B*** *)* Dose-dependent effects on R^t^ after infection with *C. concisus* 1718 from either apical or basal side in HT-29/B6 monolayers 48 h postinfection. *(* ***C*** *)* Comparison of the effect on R^t^ of *C. concisus* isolates from oral or fecal source 48 h p.i. Isolates were grouped depending on source in “healthy” (strain no. K2 oral, K4 oral, M2 oral) and “diarrhea” (strain no. 112100 fecal & oral, 113332 fecal & oral, 115605 fecal & oral) as listed in table 1. (*n.s.* = not significant; *P<0.05; *** *P*<0.001).

As shown in Figure 1*A* the incremental decrease in R^t^, beginning 22 h after bacterial inoculation (*P*<0.05, and at 48 h: *P*<0.001; Student's *t*-test) showed the same characteristic with all strains used, including ATCC 33237. HT-29/B6 monolayers that were apically or basolaterally infected with *C. concisus* at MOIs between 10 and 100 showed a dose-dependent decrease in R^t^ after 48 h, whereas infection from the basal side revealed a stronger response in R^t^ (Figure 1*B*). All oral and fecal strains had similar effects on R^t^ as shown in Figure 1*C*.

**Figure 2:**
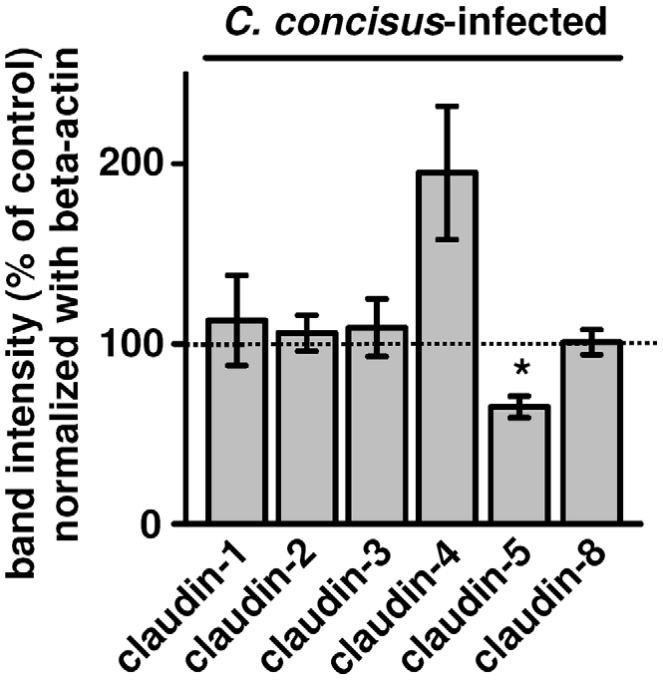
Effects on tight junction proteins. Western blot analysis on the expression of tight junction proteins in HT-29/B6 cells 48 h after infection with *C. concisus*. Densitometry on claudin-1 to -4 and -8 showed no significant difference to control values. Solely claudin-5 expression was reduced with *P<0.05; each n = 9. HT-29/B6 cells were infected with either oral or fecal strains *C. concisus* K2 oral, K4 oral, M2 oral, 75775 fecal, 86120 fecal, 118452 fecal, 119100 fecal, 1718 fecal or ATCC 33237.

The expression of TJ proteins claudin-1 to -3, -8, occludin and zonula occludens protein-1 (ZO-1) was not changed 48 h postinfection. Only claudin-5 showed a reduced expression level to 66±8% of the control value in whole cell lysate protein preparations (*P*<0.05, n = 9), whereas claudin-4 showed a tendency towards upregulation which did not reach statistical significance (194±37% of control, n = 9, *n.s.*) (Figure 2). ZO-1 distribution in *C. concisus*-infected cells differs only with respect to Triton-X 100 (TX)-soluble and TX-insoluble protein fractions [11], therefore we checked this feature also for the claudins. All claudins showed a slight drift from TX-insoluble to TX-soluble protein preparations. This drift of claudins out of the TJ to intracellular compartments could not be confirmed in confocal laser-scanning microscopy of immunostainings (data not shown). However, 24 h postinfection claudin-5 mRNA level was reduced to 49±16% (*P*<0.05, n = 4), suggesting expression regulation from the gene, likewise in *Arcobacter butzleri* infection [12]; a close relative, belonging to the epsilon-proteobacteria.

**Figure 3:**
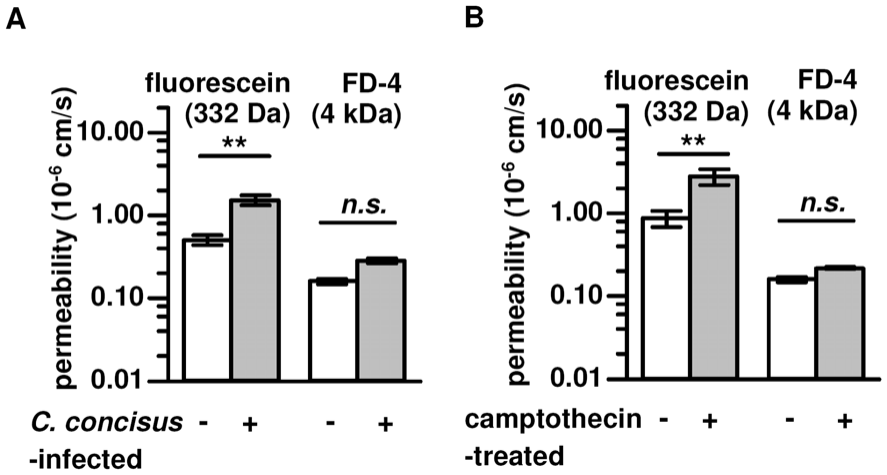
Epithelial permeability. Paracellular permeability was characterized by determining 332 Da fluorescein or 4 kDa FITC-dextran (FD-4) fluxes through *(* ***A*** *) C. concisus*-infected 48 h p.i. or *(* ***B*** *)* 48 h camptothecin-treated HT-29/B6 monolayers in Ussing chamber experiments (Student's *t*-test with *P<0.05, **P<0.01;***P<0.001; each n = 4). Infection with pooled *C. concisus* strains (oral & fecal) vs. untreated control monolayers were performed.

To further characterize epithelial barrier function, the permeability to fluorescein or FD-4 was measured. After 48 h of mucosal exposure to *C. concisus*, R^t^ of the HT-29/B6 monolayers had decreased to 60%, whereas the short-circuit current was not induced but rather slightly diminished (2.9±0.2 after *C. concisus* infection versus 3.2±0.2 µmol h^−1^ cm^−2^ in control, *n.s.*, n = 5). Concomitantly, permeability for fluorescein was increased after *C. concisus* infection (from 0.56±0.17 in control to 1.74±0.13 10^−6^ cm/s, *P*<0.01, n = 4) and permeability for FD-4 was unchanged (from 0.14±0.06 in control to 0.29±0.11 10^−6^ cm/s, *n.s.*, n = 4) (Figure 3*A*). To simulate the effects of *C. concisus* in apoptosis induction we used camptothecin (20 µg/ml) as positive control for 48 h. Camptothecin treatment of HT-29/B6 monolayers revealed similar apoptotic effects, with no changes in TJ integrity [13]. Fluorescein permeability was increased to the same extent as *C. concisus*-infected cells (from 0.84±0.33 in control to 3.09±0.38 10^−6^ cm/s, *P*<0.01, n = 4), whereas permeability to FD-4 was unchanged (from 0.15±0.06 in control to 0.21±0.03 10^−6^ cm/s, *n.s.*, n = 4, Figure 3*B*).

**Figure 4:**
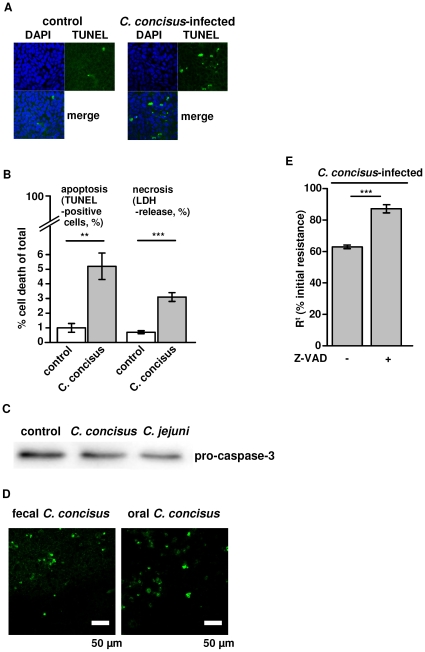
Effects of *C. concisus* on epithelial apoptosis. *(* ***A*** *)* Apoptotic effects of *C. concisus* on confluent HT-29/B6 cells visualized with TUNEL staining and fluorescence microscopy 48 h after infection, the number of cell rosette formations was increased, condensed or fragmentized nuclei were visible, and a more generalized loss of cells was observed. *(* ***B*** *)* After 48 h of incubation with *C.concisus* TUNEL staining of confluent HT-29/B6 monolayers revealed an increase in the number of apoptotic cells and LDH release was measured as a sign of necrosis induction (Student's *t*-test with **P<0.01; ***P<0.001; each n = 5). *(* ***C*** *)* Increased apoptosis was indicated by caspase-3 activation 48 h after infection. Band intensity of pro-caspase-3 (35 kDa) was significantly diminished (*P*<0.05, n = 7). *C. jejuni* ATCC 33560 served as positive control. *(* ***D*** *)* After 48 h of incubation with *C. concisus* from oral or fecal source apoptotic cell number was equal in TUNEL staining of confluent HT-29/B6 monolayers (*n.s.*, n = 5). *(* ***E*** *)* For verification of *C. concisus*-induced apoptosis effects on barrier function, HT-29/B6 monoalyers were pre-treated with 50 µM Z-VAD (N-benzyloxycarbonyl-Val-Ala-Asp-fluoromethyl-ketone), a caspase inhibitor that completely blocks caspase-mediated cell death. Transepithelial resistance was partially although not completely reconstituted by Z-VAD. Isolates were grouped depending on source in “oral” and “fecal” as listed in table 1.

**Figure 5:**
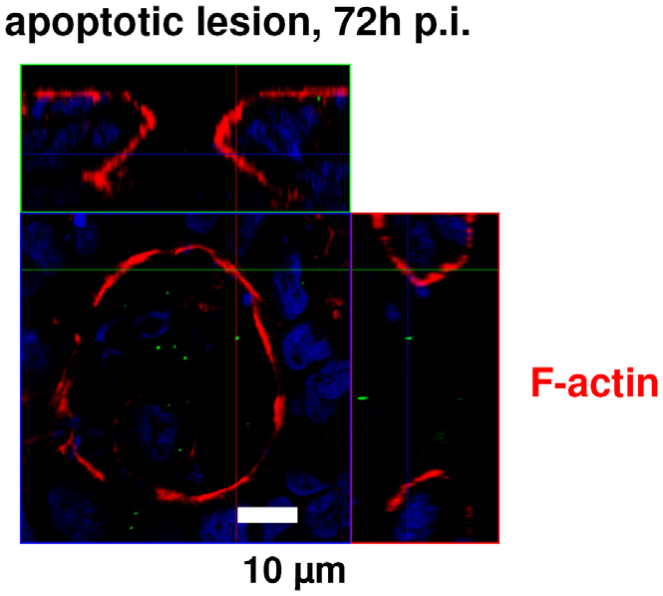
Apoptotic leaks. Confocal laser-scanning microscopy (*Z*-axis scans, *XY* plane) of an apoptotic lesion in HT-29/B6 monolayers infected with *C. concisus*. Cytoskeletal F-actin is marked red by Phalloidin-Alexa-Fluor^594^, the blue staining with DAPI ( = 4′-6-diamidino-2-phenylindole dihydrochloride) marks the nuclei of epithelial cells and *C. concisus* is marked green by anti-*Campylobacter* antibody. Details are presented in Figure S1. In controls, superficial single cell lesions were closed within 16 min, accompanied by formation of an actin ring (“*purse-string*”) that is essential for restitution. Recovery of apoptotic lesions, induced by *C. concisus*, seems unaffected as the purse-string actin formation is clearly visible.

**Figure 6:**
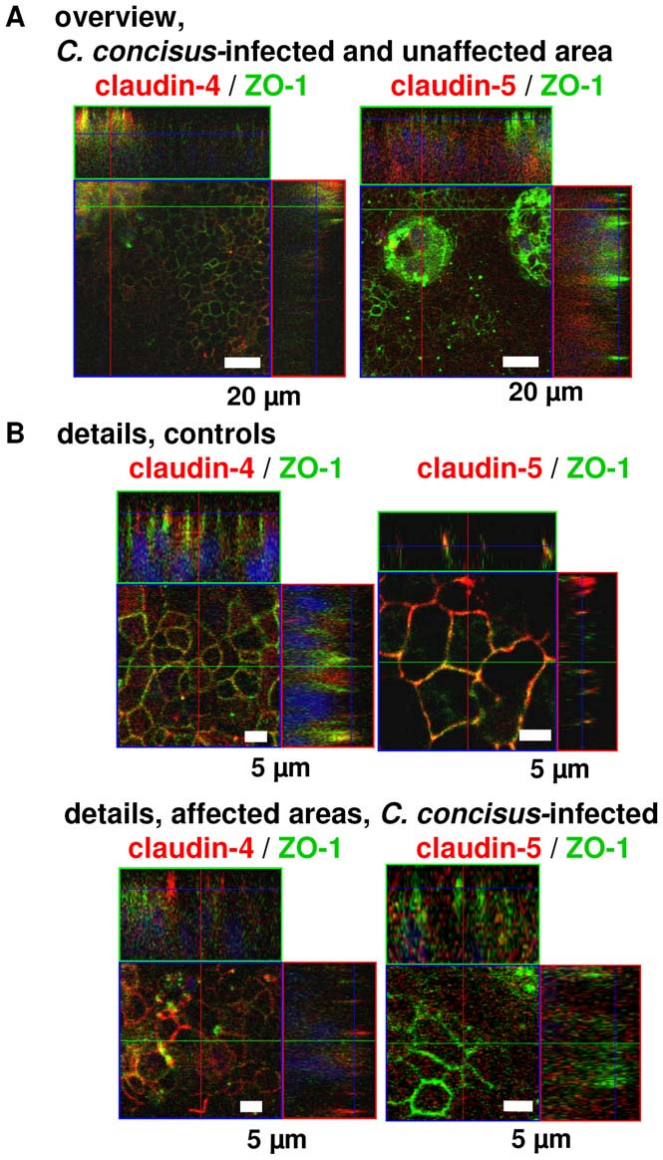
Apoptosis and tight junctions. Confocal laser-scanning microscopy of HT-29/B6 monolayers revealed patchy distributed areas with tight junction changes and apoptotic events as well as largely unaffected areas in *(* ***A*** *)* overviews with low magnification and *(* ***B*** *)* details with higher magnifcation. Immunostaining with green signal for ZO-1; and claudin-4 and -5 marked red; merge appears yellow. Nuclei are stained blue with DAPI.

The barrier dysfunction after infection could on the one hand result from changes or disruption of TJ strands but on the other hand also from a loss of epithelial cells by apoptosis or necrosis induction. Apoptosis as examined histologically in TUNEL staining indicated an increased apoptotic ratio in *C. concisus*-infected cells (Figure 4*A*). Furthermore, LDH release was determined as marker for cytotoxicity. LDH release was elevated to 3.1±0.3% versus 0.7±0.1% in control (*P*<0.001; n = 5) suggesting cytotoxic effects caused by *C. concisus* (Figure 4*B*). After 48 h infected monolayers showed a 5-fold increase in epithelial apoptosis (5.2±0.9% vs. 1.0±0.3% in control; *P*<0.01; n = 5). In Western blots, pro-caspase-3 band intensity decreased to 70±2% in infected monolayers (*P*<0.05, n = 7, Figure 4*C*), but the caspase-3 activation was weaker when compared to the effects of *C. jejuni* ATCC 33560, where pro-caspase-3 was decreased to 52±6% of control values (*P*<0.01, *C. concisus* vs. *C. jejuni*, n = 4), while the apoptotic ratio was equal between *C. concisus*- and *C. jejuni*-infected cells (4.6±0.3%, *n.s.* vs. *C. concisus*, n = 4). Fecal and oral strains did not differ in their ability to induce apoptosis in HT-29/B6 cells with 4.8±1.4% by oral strains vs. 4.7±0.5% apoptosis by fecal strains (*n.s.*, n = 5; Figure 4*D*). Pre-treatment with the apoptosis inhibitor Z-VAD showed a reduced loss of R^t^ in *C. concisus*-infected cells (at 48 h, *C. concisus* 112100 fecal & oral; without Z-VAD 63.5±1.5% vs. with Z-VAD 86.7±2.9% of initial resistance, *P*<0.001; n = 8; Figure 4*E*). Moreover, when we prolonged the incubation time to 72 h, we detected *C. concisus* next to or in epithelial lesions with restitutional purse-string formation in confocal laser-scanning micrographs (Figure 5 and Figure S1). Also, patchy distributed apoptotic areas with TJ changes were found beside largely unaffected areas in each monolayer (Figure 6). As an example it is shown in Figure 6 that claudins of infected monolayers were not clearly redistributed towards intracellular compartments.

## Discussion

As a main finding, we demonstrated that *C. concisus* was able to induce barrier dysfunction in confluent HT-29/B6 cells from apical but even more from the basolateral compartment. Although controversially discussed [9], [11], also oral *C. concisus* strains, even from healthy carriers, had the ability to impair epithelial barrier function. Man and coworkers demonstrated the polar flagellum of *C. concisus* to be involved in attachment and invasion of Caco-2-cells [11]. We found similar invasion abilities for our *C. concisus* strains in HT-29/B6 as well as Caco-2-cells (data not shown). Recently, it was also reported that *C. concisus* translocated across the Caco-2 monolayers and suggested that the concomitant increase in permeability for 44 kDa horseradish peroxidase (HRP) 6 h postinfection was through a paracellular pathway [11]. In contrast to this, Söderholm and coworkers reported that HRP fluxes through the epithelium were due to a transcytotic pathway via HRP-containing endosomes [14], at least in intact epithelial layers. Therefore it might be that *Campylobacter* invasion is rather correlated with the transcytosis ratio (macropinocytosis) and not with a paracellular disruption, but perhaps a spatial TJ redistribution is accelerated by this process. However, the causal link between the barrier dysfunction of *C. concisus*-infected cells and a pathomechanism via the paracellular pathway by apoptosis induction and/or TJ affection ( = leak-flux dysfunction) is supported by our data. We investigated the protein expression of barrier-forming claudins. The claudin protein family is most important for epithelial barrier function even though not all claudins have sealing properties. We did only find a weak influence on claudin-4 and -5 and no effect on claudin-1 to -3, -8, occludin or ZO-1. This is contrary to other enteric pathogens with a stronger impact on TJs, e.g. enteropathogenic *Escherichia coli* or *Arcobacter butzleri* [12]. Our results did not display an overall alteration of the TJ. The only reduction in the sealing TJ protein claudin-5 was rather weak, thus it seems likely that the TJ is functionally only moderately affected. However, infected epithelial cells were destroyed, as demonstrated by a release of LDH and an increase in apoptotic events. The most important result of our study was that both, oral and fecal strains induced apoptotic leaks leading to an increase in epithelial permeability to fluorescein. Pre-treatment with the apoptosis inhibitor Z-VAD showed a reduced loss of R^t^ over 48 h and confirms the impact of *C. concisus*-induced apoptosis on barrier function. Interestingly, a similar experiment with *A. butzleri*, which shows more impact on the TJ and also an effect on apoptosis, results in a partial reconstitution of *A. butzleri*–induced barrier dysfunction by inhibition of apoptosis with Z-VAD. This enabled us to distinguish the barrier impairment caused by TJ disruption from apoptotic influences [12]. The comparison of tracer fluxes in *C. concisus*- or camptothecin-exposed monolayers revealed an increase in 332 Da fluorescein permeability but no difference for 4 kDa dextran in the same time period with both treatments, suggesting that apoptosis induction could be the main cause of *C. concisus*-induced barrier dysfunction. This result correlates to previous results of our group that revealed increased permeability in apoptotic monolayers to the same characteristic molecule size cut-off at 4 kDa. In these experiments camptothecin-induced apoptosis in HT-29/B6 monolayers showed also an increased permeability towards intermediate molecule size of 344 Da (H^3^-lactulose) and no change for 4 kDa tracer molecules (H^3^-polyethylene glycol) [13]. Thus, the apoptosis induction by *C. concisus* seems to be the prominent feature of infection. The induction of epithelial cell death could be caspase-dependent but also caspase-independent as already shown for *C. jejuni* [15]. As shown for the hemolytic toxin aerolysin from *Aeromonas hydrophila*, the pore-forming activity of aerolysin interferes with restitutional purse-string formation [16] of small epithelial lesions via MLCK-dependent actomyosin constriction [17]. However, this rapid inhibitory consequence on epithelial recovery was not the case with *C. concisus*. Here, purse-string formation was still visible even 72 h p.i. The hemolytic activity of *C. concisus* is low, compared to typical hemolytic bacteria, but all *C. concisus* strains used in our study showed some hemolytic activity (data not shown), measured by the contact hemolysin assay [7].

Our findings suggest *C. concisus* as an enteric pathogen, characterized by defined spatial interactions with human epithelial cells. We demonstrate that both oral and fecal *C. concisus* strains induce impairment of the intestinal barrier function. The epithelial necrosis and apoptosis induction are mechanisms that can contribute to a leak-flux type of diarrhea. Also, this leads to a higher amount of antigens passing the mucosa that can accelerate inflammatory processes. Campylobacteriosis is a well-known zoonosis, mostly caused by *C. jejuni* through contaminated food. In our study eight patients with diarrhea had *C. concisus* in their feces, whereas three healthy individuals had *C. concisus* only in saliva samples. Investigations on biofilm formation in the oral cavity and on the ecological niche of *C. concisus* would be worthwhile also for research on the route of transmission (oral-oral and/or fecal-oral). Humans might be the main reservoir for *C. concisus* infection, as known from *Helicobacter pylori* infection. There is a need for extensive investigation of veterinary samples in search for possible additional reservoirs of *C. concisus*. Furthermore, there is an urgent need for clinical data with a proper follow-up period to ascertain, if this emerging pathogen is an etiologic factor in the development of chronic diarrhea or inflammatory bowel diseases as recently proposed for *Salmonella* or *Campylobacter* enteritis [18].

## Materials and Methods

### Isolation and growth condition of *C. concisus*

Six oral and eight fecal strains of *C. concisus* were selected for the study. Fecal strains were sampled from eight patients with diarrhea (age 2–63 years). Three of the patients were also asked to deliver saliva samples from which *C. concisus* was cultivated. The remaining three oral strains were cultivated from saliva samples of three healthy individuals (age 25–31 years) who had no *C. concisus* in their fecal samples and no history of gastrointestinal disease (Table 1). This study adhered to the Declaration of Helsinki, and ethics approval for research was obtained from The Ethics Committee for Region Nordjylland, Denmark (Den Videnskabsetiske Komité for Region Nordjylland; N-20080056). All patients who participated in the investigation signed written informed consent forms. For children written informed consent forms were signed by their parents. All strains were analyzed at the Department of Clinical Microbiology, Aalborg Hospital, Aarhus University Hospital, Denmark. *C. concisus* was isolated by the filter technique on 5% horse blood agar plates, containing 1% yeast extract (SSI Diagnostica, Hillerød, Denmark), and incubated at 37°C in a microaerobic atmosphere with 3% hydrogen [19]. Final confirmation was obtained through a species-specific real-time PCR based on the cpn60 gene, as described elsewhere [20]. No other pathogen was isolated in the patients' fecal samples. All strains were stored in nutrient beef broth with 10% glycerin (SSI Diagnostica) at −80°C until use. Prior to experimental infection of epithelial cell cultures, bacteria were grown on 5% horse blood agar plates, containing 1% yeast extract (SSI Diagnostica) at 37°C in microaerobic atmosphere with 3% hydrogen for 48 h. Then bacteria cells were transfered to liquid culture medium RPMI 1640 (PAA Laboratories, Pasching, Austria) and cultured again for 48 h to log-phase. Bacterial density was counted microscopically and adjusted to a choosen multiplicity of infection (MOI). The *C. concisus* reference strain ATCC 33237 from human gingival sulcus was cultured in the same way.

**Table 1:** Proband data at time of *Campylobacter concisus* isolation.

Age	Sex	Clinical characteristic	Source	Isolate no.
25	Female	Healthy	oral	K2
31	Female	Healthy	oral	K4
26	Male	Healthy	oral	M2
29	Male	Crohn's disease	fecal	75775
60	Male	Diarrhea	fecal	86120
19	Female	Crohn's disease	fecal	118452
63	Female	Collagenous colitis	fecal	119100
2	Female	Bloody diarrhea	fecal	1718
36	Female	Crohn's disease	fecal & oral	112100
57	Female	Diarrhea	fecal & oral	113332
42	Male	Diarrhea	fecal & oral	115605

### Epithelial cell culture

HT-29/B6 cells represent an established cell model for studies on bacterial infection [12], [17], [21]. HT-29/B6 cells were established and described by Kreusel et al. in 1991, as a stable and highly differentiated subclone derived from wild-type HT-29 cells by glucose deprivation [22]. The cells were cultured in RPMI cell culture medium (RPMI 1640, PAA Laboratories, Pasching, Austria) as described previously [22]. Cells were grown to confluence on polycarbonate filter supports (Millicell-PCF™, effective membrane area 0.6 cm^2^, pore size 3 µm, Millipore, Billerica, MA., USA), and formed high-ohmic epithelial monolayers with a transepithelial electrical resistance (R^t^) between 500 and 800 Ohm⋅cm^2^ eight days after seeding. In parallel to the infection with all *C. concisus* strains, three types of controls were performed: (i) without adding bacteria, (ii) with addition of heat-inactivated *C. concisus* or *C. concisus* lysates (60 min at 75°C) and (iii) with *Escherichia coli* K12.

### Electrophysiological studies

In order to investigate the effect of the oral and fecal *C. concisus* strains on epithelial integrity, R^t^ of polarized HT-29/B6 monolayers was measured after infection with chopstick electrodes (Word Precision Instruments, Sarasota, FL., USA) combined with an ohmmeter (manufactured by D. Sorgenfrei at the Institute of Clinical Physiology, Charité, Berlin) in the cell culture dish under sterile conditions at 37°C. Bacteria were added to the apical compartment of confluent HT-29/B6 cell monolayers to yield an initial concentration of 2×10^7^ cfu/ml equaling a MOI of 20. Infected HT-29/B6 cell monolayers were mounted into modified Ussing-type chambers (manufactured by the Institute of Clinical Physiology, Charité, Berlin) [22] after 48 h. Short circuit current (Isc) and R^t^ were determined by using a computerized automatic clamp device (Fiebig Hard & Software, Berlin, Germany). Infections were done in an atmosphere with 5% CO_2_ in ambient air in favor for the HT-29/B6 cell line. For an additional assay concerning dose-dependent effects, monolayers were apically or basolaterally infected with *C. concisus* at MOIs between 10 and 100.

### Epithelial permeability

Unidirectional tracer flux studies were performed from the apical to the basolateral compartment under short-circuit conditions in Ussing chambers with fluorescein (332 Da) and fluorescein isothiocyanate-labeled 4 kDa dextran (FD-4) (Sigma, St. Louis, MO., USA) according to previous described protocols [23], [24]. Medium in the basolateral compartment was initially free of dyes. At specific intervals, medium was withdrawn from the basolateral chamber and fluorescence was measured in a spectrophotometer (Tecan Infinite M200, Durham, NC., USA). Permeability was calculated from flux over concentration difference. Camptothecin-treated monolayers were also investigated by tracer flux measurements, in order to quantify barrier disturbance by apoptosis induction.

### Western blot analysis

TJs play a key role in epithelial integrity and may be affected during infection. We investigated the TJ protein expression of barrier-forming claudins after infection with *C. concisus*. Western blot analysis TJ proteins were quantified by densitometry. Immunoblots were performed and analyzed as described elsewhere [25]. Detergent-soluble protein fractions were prepared from infected monolayers. The following antibodies were used: anti-ZO-1, anti-occludin (1∶2000; Zymed, San Francisco, CA., USA), anti–claudin-1–5 and -8 (1∶1000; Zymed), anti–β-actin (1∶5000; Sigma), and anti–caspase-3 (1∶1000; Cell Signaling Technology, Beverly, MA., USA).

### Immunofluorescence microscopy

To test for the integrity of the TJ meshwork, TJ proteins ZO-1, occludin, claudin-1 to 5 and claudin-8 were visualized using confocal laser-scanning microscopy (Zeiss LSM510, Jena, Germany), analyzed by Carl Zeiss LSM Image Examiner software according to prior descriptions [25]. For detection, antibodies raised against TJ proteins (1∶50; Zymed) or *Campylobacter* (Santa Cruz Biotechnology Inc, Santa Cruz, CA. USA) were used. For cytoskeletal F-actin, staining with Phalloidin-Alexa-Fluor^594^ (Sigma) was performed.

### Cytotoxicity. LDH release assay

Lactate dehydrogenase (LDH) release from HT-29/B6 cells was measured according to the method of Madara and Stafford [26]. ***TUNEL staining*** was performed according to manufacturer's protocol (In-situ Cell Death Detection Kit, Roche, Mannheim, Germany). Stained apoptoses were counted microsopically. We investigated the properties of oral and fecal *C. concisus* isolates in apoptosis induction by TUNEL staining and Western blotting on caspase-3 activation.

### Invasion assay

Bacteria were harvested from agarplates (5% horse blood agar +1% yeast extract; SSI Diagnostica) after 48 h and equally diluted in 3 ml RPMI-media, vortexed, and incubated another 48 h in a 37°C microaerobic atmosphere with 3% hydrogen. Monolayers were infected from the apical side with a MOI of 10 and treated (i) with gentamicin (100 µg/ml) prior to infection, (ii) with gentamicin after 2 hours of incubation, or (iii) without gentamicin. Bacterial supernatants were proven for bacterial count. The monolayers were then washed three times with PBS and flooded with 300 µl 0.5% Triton-X 100. *C. jejuni* ATCC 33560 was used as positive control.

### Quantitative Realtime-PCR

Total RNA was obtained from HT-29/B6 cells by phenol-chloroform extraction and complementary DNA (cDNA) was synthesized by reverse-transcription PCR with High-Capacity cDNA Archive Kit (Applied Biosystems, Mannheim, Germany) with random primers. Real-time PCR was performed according to manufacturer's instructions with an ABI-7900HT PCR device using the TaqMan Gene Expression Assay (no. Hs00533949_s1; claudin-5) with FAM dye-labeled primers. Glyceraldehyde 3-phosphate dehydrogenase cDNA was quantified using VIC reporter dyes as endogenous control (all Applied Biosystems). Differential expression was calculated according to the 2^−ΔΔCT^ method [27].

### Statistical analysis

Data are expressed as means ± standard error of the mean (SEM). Statistical analysis was performed using the Student *t*-tes*t*. *P*<0.05 was considered significant.

## Supporting Information

Figure S1 **Apoptotic leaks.** 360° view of the confocal laser-scanning micrograph of an apoptotic lesion in HT-29/B6 monolayers infected with *C. concisus*, based on Figure 5 (*Z*-axis scans, *XY* plane). Cytoskeletal F-actin is marked red by Phalloidin-Alexa-Fluor^594^, the blue staining with DAPI ( = 4′-6-diamidino-2-phenylindole dihydrochloride) marks the nuclei of epithelial cells and *C. concisus* is marked green by anti-*Campylobacter* antibody. In controls, superficial single cell lesions were closed within 16 min, accompanied by formation of an actin ring (“*purse-string*”) that is essential for restitution. Recovery of apoptotic lesions, induced by *C. concisus*, seems unaffected as the purse-string actin formation is clearly visible.
(MPG)
